# Supplementary figures and images for: Kombucha Tea-associated microbes remodel host metabolic pathways to suppress lipid accumulation
Source: PLoS Genet. 2024 Mar 28;20(3):e1011003. doi: 10.1371/journal.pgen.1011003 (PMC10977768; doi:10.1371/journal.pgen.1011003)

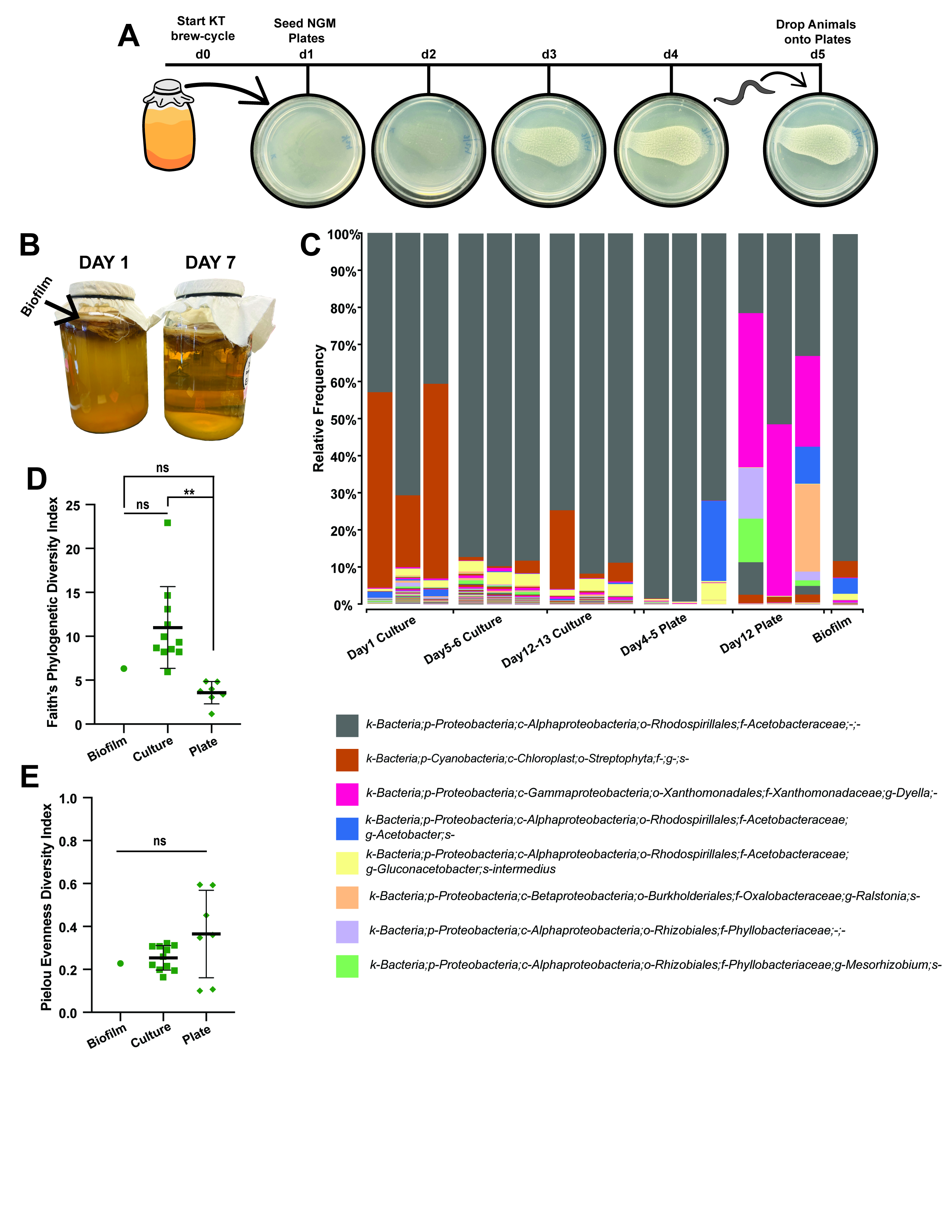

Supplement: S1 Fig — (A) Images of NGM worm plates seeded with a KTM lawn. The preparation starts at day 0 when a new KT brew cycle is initiated, the microbes are seeded on day 1, and incubated at room temperature to day 5 before the KTM plates are used. (B) Representative photos of KT brews at day 1 and day 7 of fermentation. The KTMs are extracted from the culture at day 1 and plated. (C) A comprehensive view of 16S rDNA sequencing results of the KT microbes from fermenting Kombucha culture, seeded NGM plates, or the pellicular biofilm from the Kombucha culture. The plot shows the frequency of each species (8 most abundant microbes displayed; a complete list can be found in S1 Table). (D) A plot of Faith’s phylogenetic diversity index showing the difference in α-diversity between the indicated samples (**, p<0.01, one-way ANOVA). (E) The Pielou Evenness Diversity Index, measuring the microbial diversity and species richness in the indicated samples (ns, not significant, one-way ANOVA). Raw data underlying panels C-E can be found in S7 Data. (TIF) [file pgen.1011003.s001.tif]

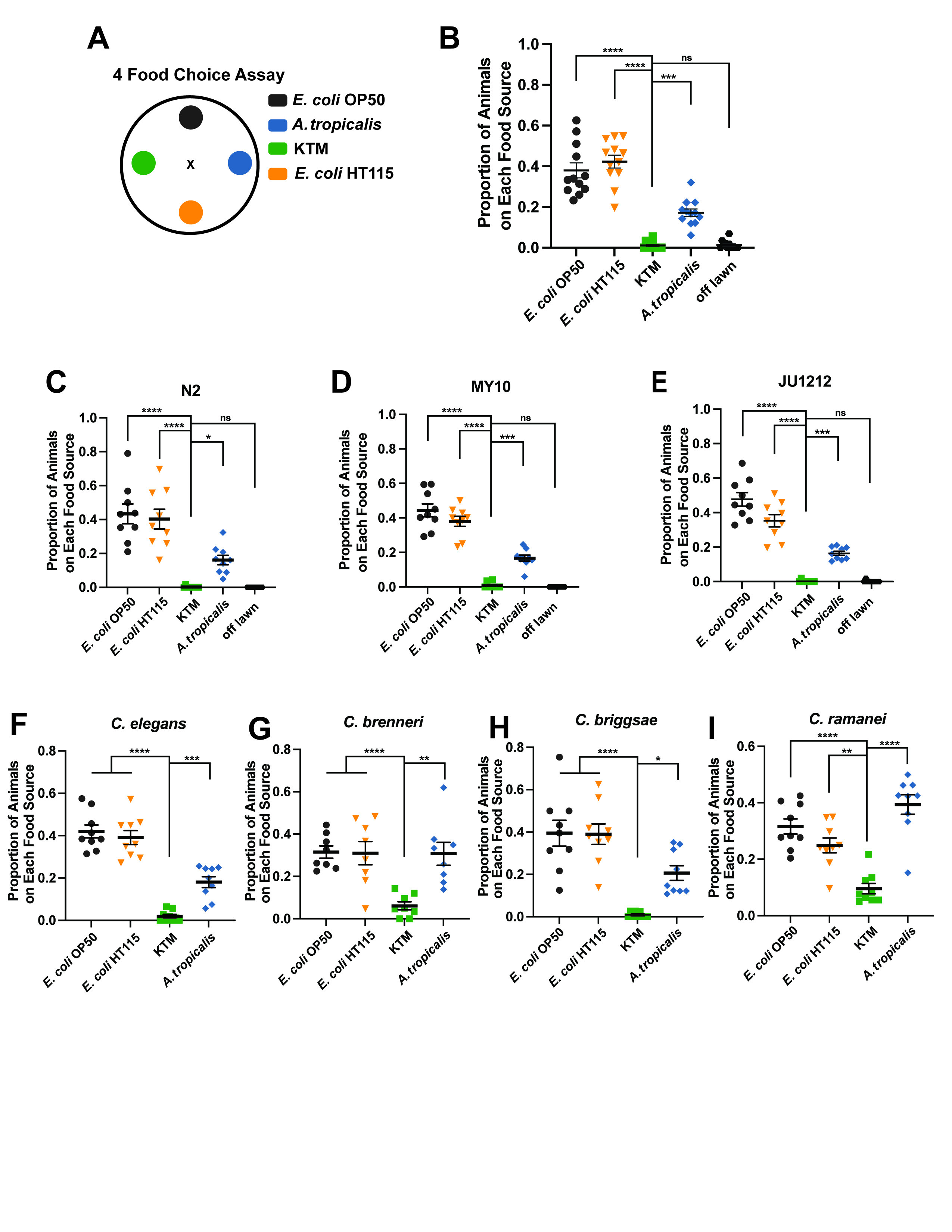

Supplement: S2 Fig — (A) A schematic depicting the food choice assay. (B) The portion of wild-type N2 animals at the L4 stage on each food source 48 hours after dropping L1s (n>200/trial, 3 biological replicates). (C-E) Food choice assays for the N2, MY10, and JU1212 C. elegans strains scored at the L4 stage (48h post L1 drop, n>150/trial, 3 biological replicates). (F-I) The portion of L4 stage worms on each food source at 48h post L1 drop for the N2 C. elegans, PB2801 C. brenneri, AF16 C. briggsae, and PB4641 C. remanei strains (n>75/trial, 3 biological replicates). All food choice data are plotted as the mean ± SEM. All food choice assays include n>150 animals per replicate and the data are plotted as the mean ± SEM (****, P<0.0001, ***, P<0.001, **, P<0.01, *, P<0.05, ns, not significant; one-way ANOVA). Raw data underlying panels B-I can be found in S8 Data. (TIF) [file pgen.1011003.s002.tif]

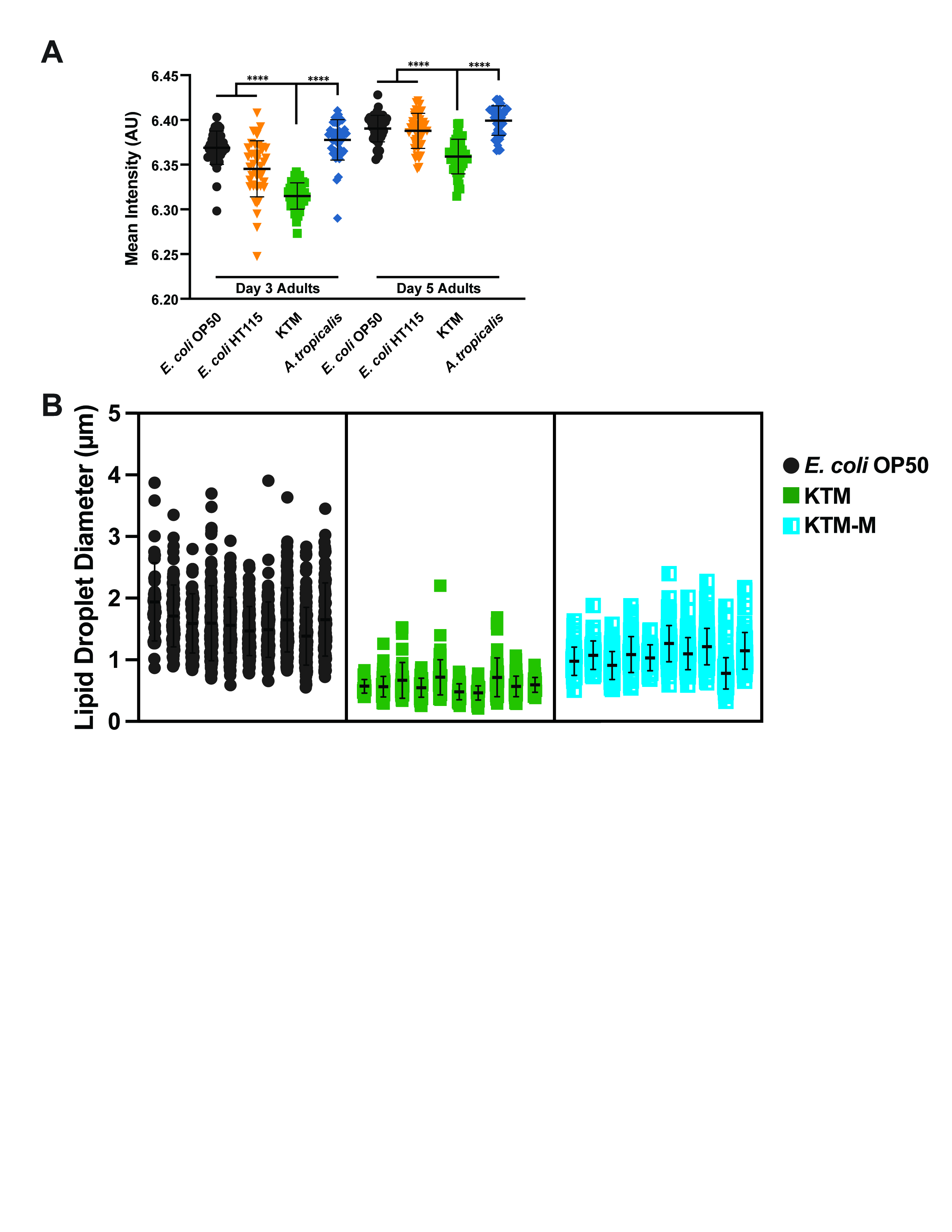

Supplement: S3 Fig — (A) Quantification of day 3 and day 5 adults stained with Oil Red O (mean ± SD, ****, P<0.0001, one-way ANOVA). (B) Measurements of individual lipid droplet sizes measured across ten individuals consuming E. coli OP50, KTMs, or KTM-Mix (mean ± SD, n = 10 animals/trial, 2 biological replicates). The distribution of lipid droplet sizes is similar across individuals fed the same diet. Raw data underlying panels A and B can be found in S9 Data. (TIF) [file pgen.1011003.s003.tif]

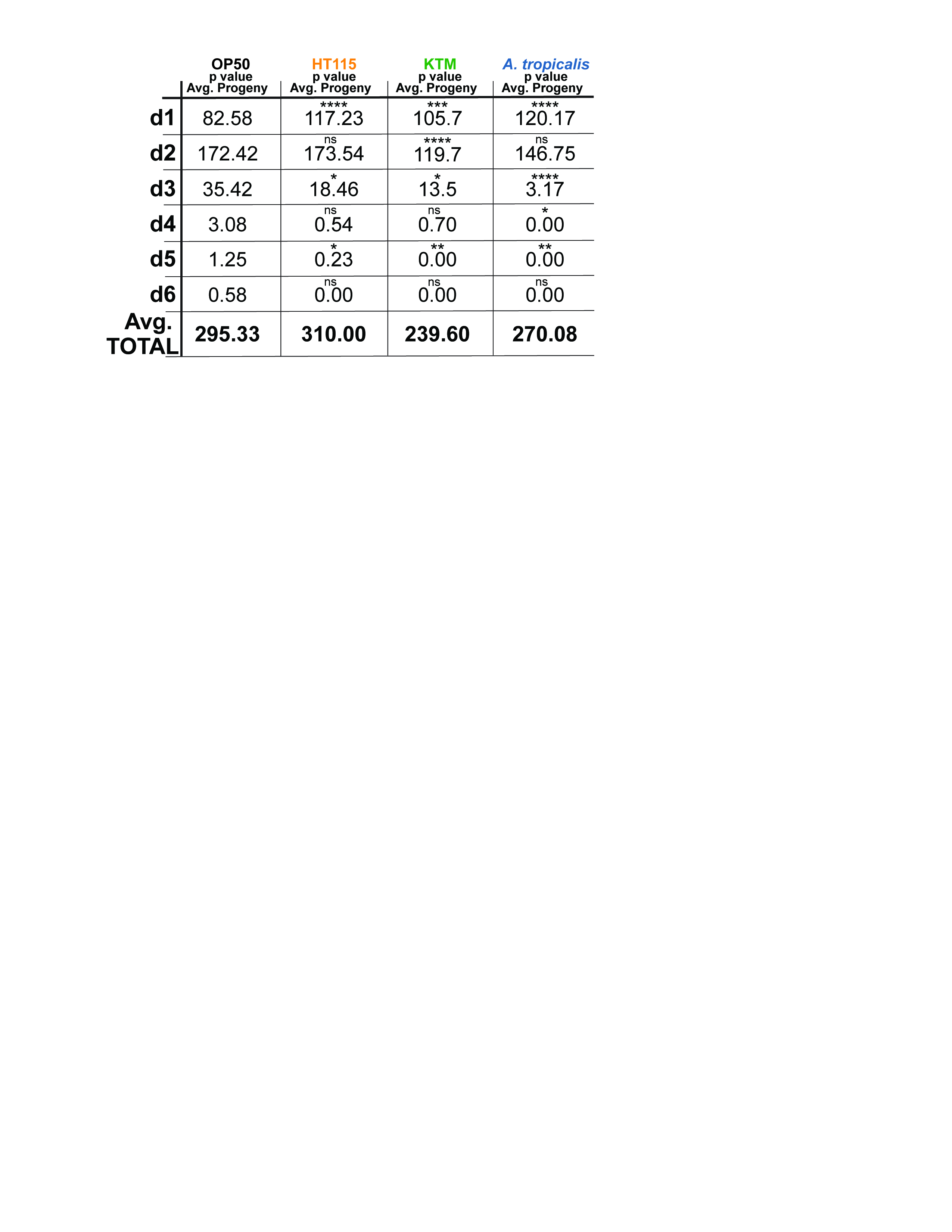

Supplement: S4 Fig — A table displaying the average progeny laid per day of the reproductive period demonstrates that KTM-fed animals exhibit a similar egg laying rate relative to E. coli OP50-fed animals (mean, ****, P<0.0001, ***, P<0.001, **, P<0. 01, *, P<0.05, ns, not significant, T-test). Raw data underlying the figure can be found in S10 Data. (TIF) [file pgen.1011003.s004.tif]

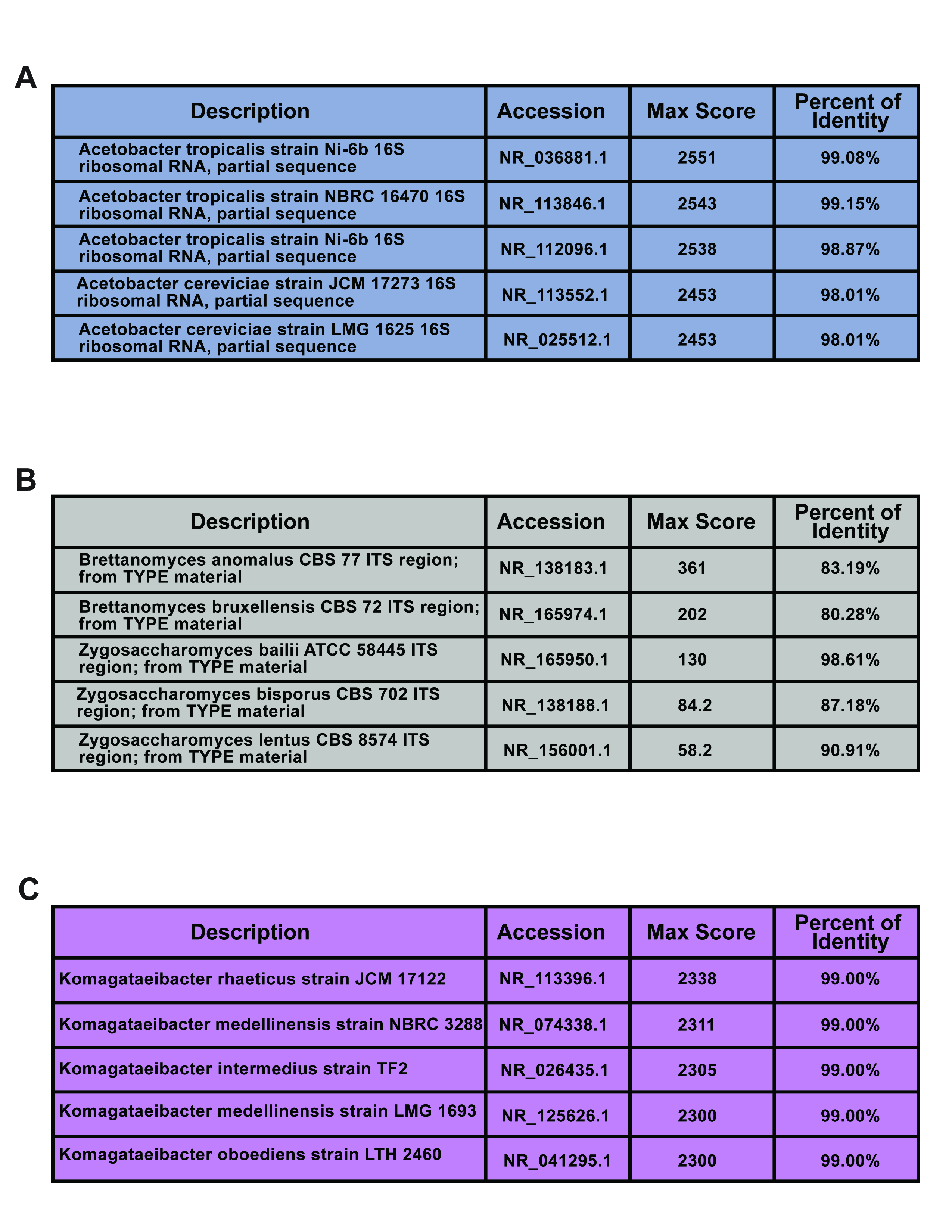

Supplement: S5 Fig — Results from 16S rDNA sequencing of the isolated bacterial KTMs indicate that (A) A. tropicalis and (C) a member of the Komagataeibacter genus are components of our Kombucha culture. (B) Sequencing of the ITS region of the KTM yeast isolate revealed that the strain belongs to the Brettanomyces or Zygosaccharomyces genus. Raw data underlying panels A-C can be found in S1 Table. (TIF) [file pgen.1011003.s005.tif]

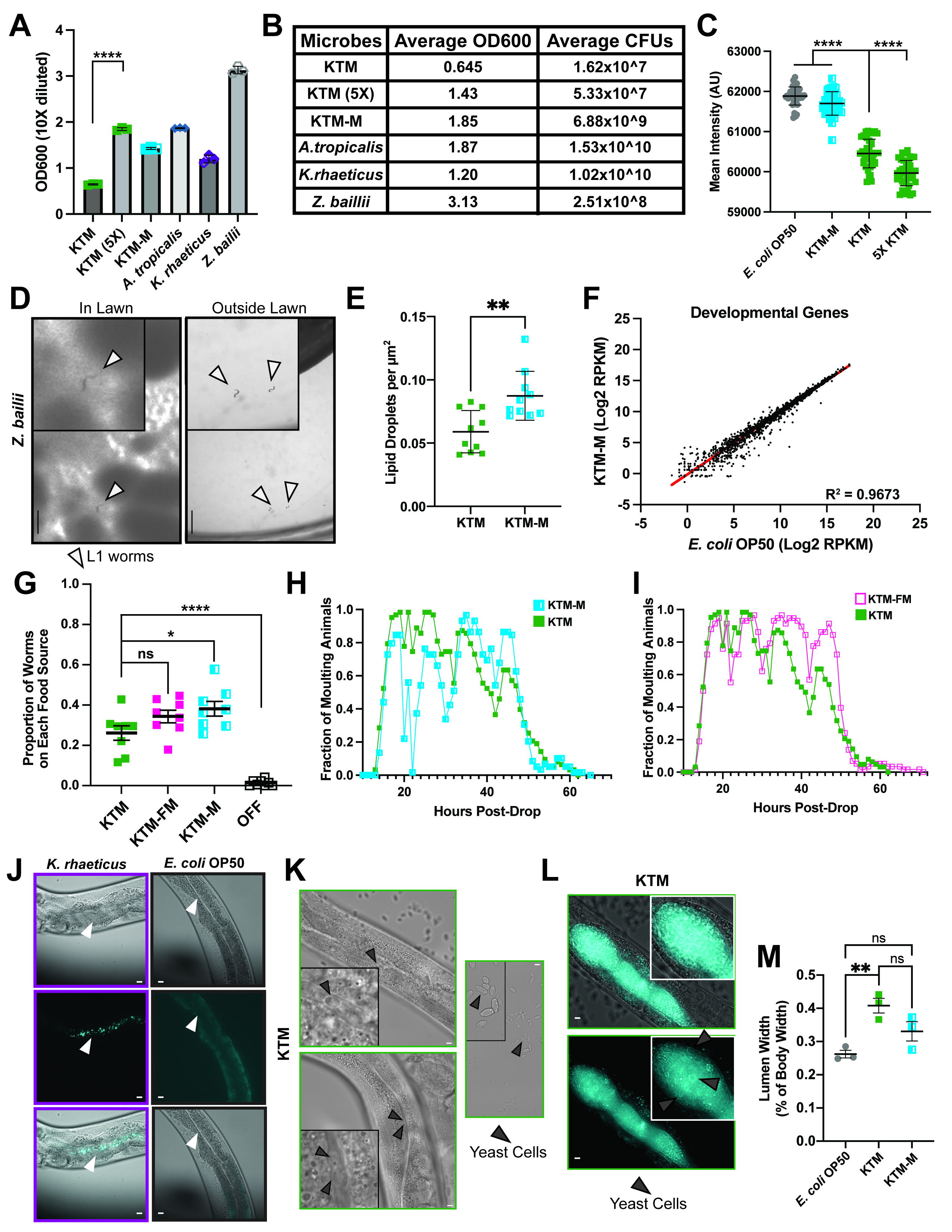

Supplement: S6 Fig — (A-B) Measurements of the microbial concentrations in each of the indicated microbial mixes or single microbial cultures (mean ± SEM, ****, P<0.0001, one-way ANOVA). (C) Oil Red O staining of day 1 adult animals fed an E. coli OP50, KTM-Mix, or KTMs diet, as well as a 5X concentrated version of the KTM diet (mean ± SD, ****, P<0.0001, one-way ANOVA). Increasing the concentration of KTMs decreases lipid storage. (D) Representative images of animals off and on a lawn of Z. bailii yeast 72 hours post L1 drop, which shows that animals fail to develop when consuming a Z. bailii diet (worms are indicated with white arrow heads; scale bar, 500 μm). (E) Lipid droplet density measurements with each datapoint representing the number of lipid droplets per μm2 for the last two intestinal cells of animals consuming a KTM or KTM-M diet (the KTM data are also shown in Fig 2H; mean ± SD, **, P<0.01, T-test). (F) A scatter plot comparing the expression of 2,229 developmental genes in animals fed E. coli versus KTM-M as determined by mRNA-Seq (RPKM, reads per kilobase of transcript per million mapped reads). A linear regression analysis and the corresponding R2 value is reported. (G) A choice assay showing the portion of wild-type N2 animals at the L4 stage on the indicated food sources 48 hours after dropping L1s (n>200/trial, 3 biological replicates; mean ± SEM, ****, P<0.0001, *, P<0.05, ns, not significant, one-way ANOVA). (H-I) The developmental rate of animals expressing a Pmlt-10::GFP-PEST reporter when fed a KTM, KTM-Mix, or a KTM-FM diet. Synchronized L1 worms were reared at 20°C for ~72 hours and scored hourly. (J) Representative images of animals consuming K. rhaeticus or E. coli OP50 after staining with Calcofluor White, which selectively labels intestinal microbes producing chitin or cellulose (white arrow heads indicate the intestinal lumen; scale bars, 10 μm). (K) Representative brightfield DIC images showing yeast cells in the intestine of animals consuming KTMs and y [file pgen.1011003.s006.tif]

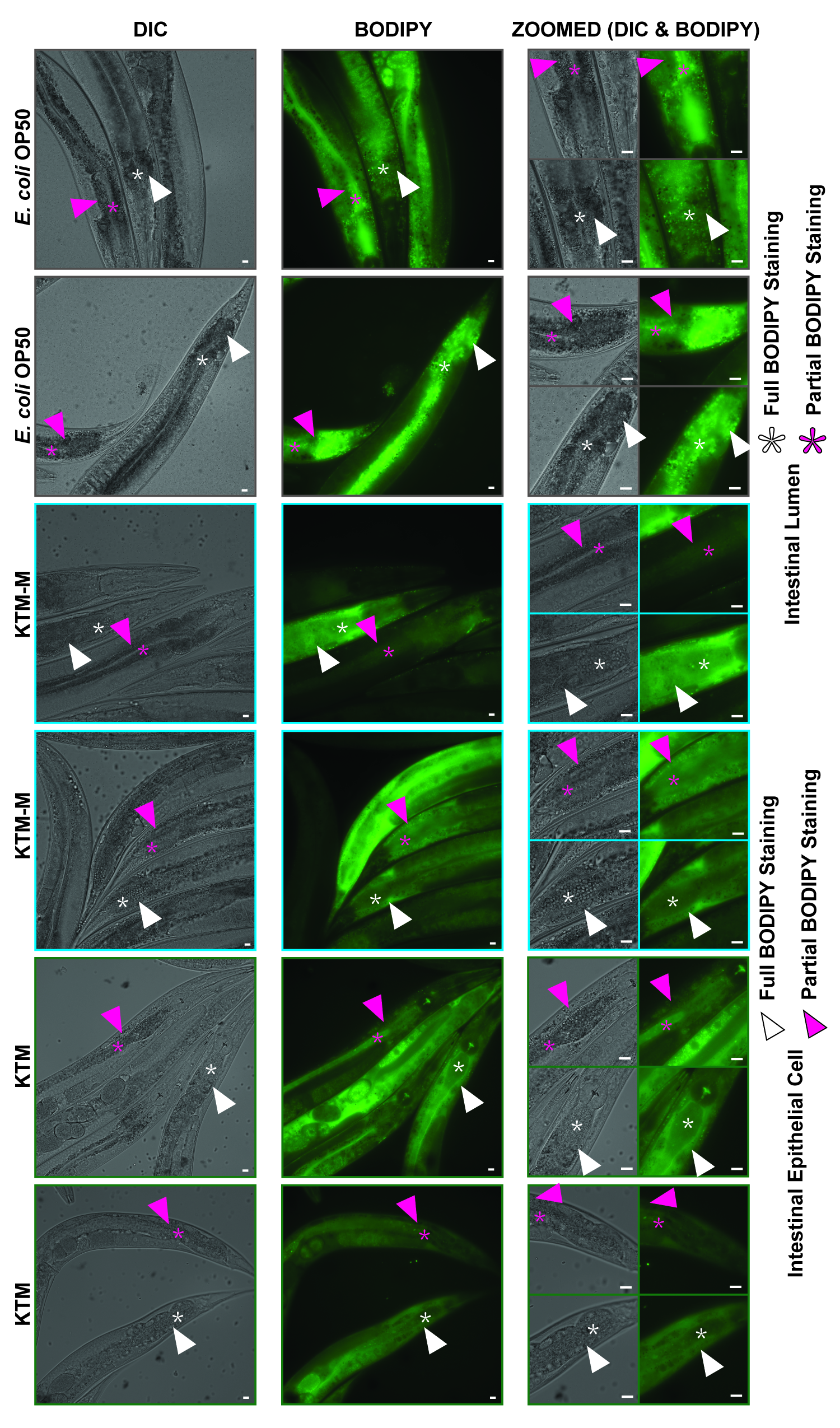

Supplement: S7 Fig — Representative DIC and fluorescence images showing C1-BODIPY-C12 absorption into the intestinal epithelial cells of animals feeding on an E. coli OP50, KTM, or KTM-M diet. The pink stars indicate BODIPY remaining in the intestinal lumen, the pink arrowheads point to partial BODIPY absorption into the intestinal cells, white stars indicate a lack of BODIPY remaining in the intestinal lumen, and white arrowheads point to fully stained cells that have absorbed BODIPY (scale bars, 10 μm). (TIF) [file pgen.1011003.s007.tif]

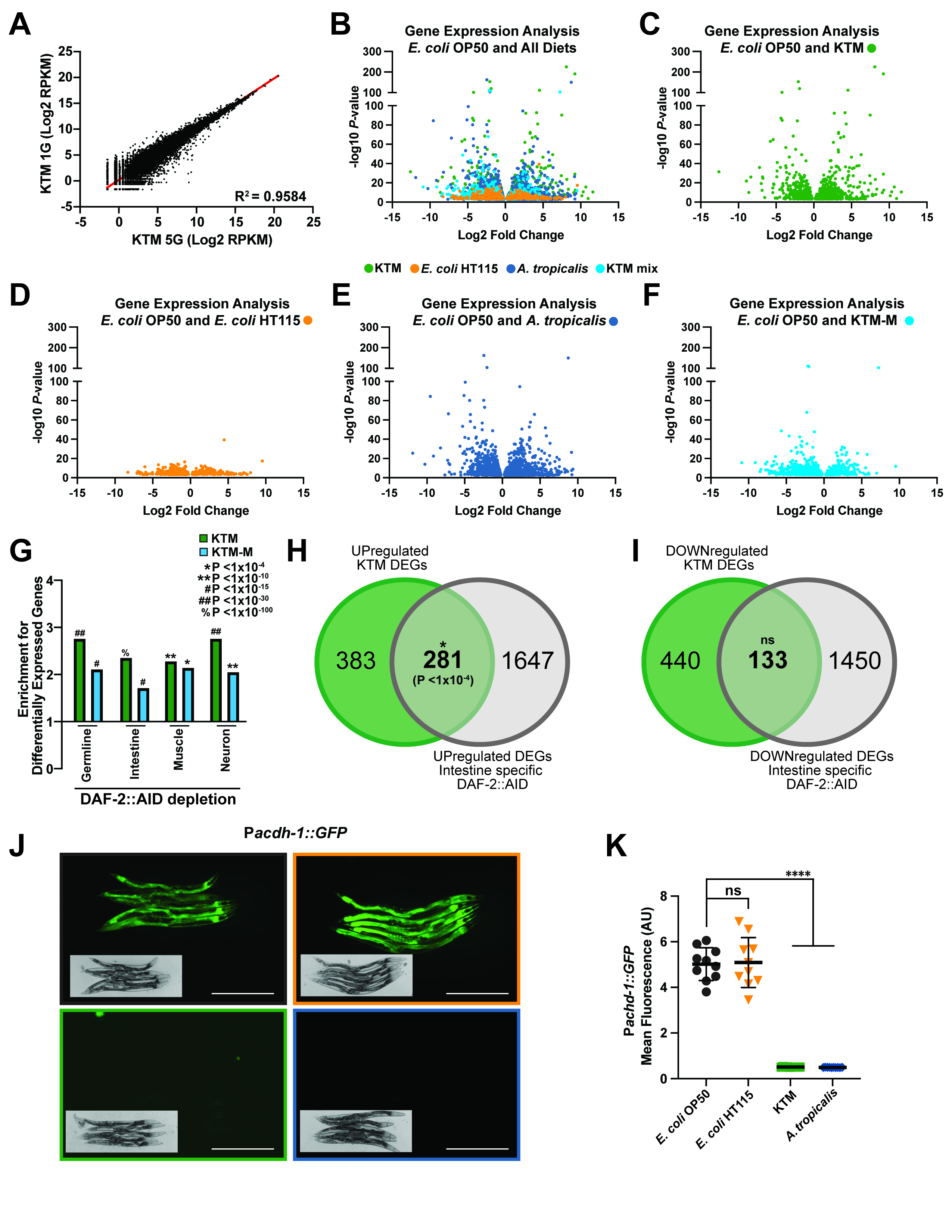

Supplement: S8 Fig — (A) A scatter plot and linear regression analysis comparing the expression of all genes in animals fed KTMs for one generation (1G) or for five generations (5G), suggesting that pervasive transgenerational epigenetic regulation of gene expression by KTMs is unlikely. (B-F) Volcano plots showing the differentially expressed genes for the indicated samples relative to the E. coli OP50 sample. (G) Enrichment (observed/expected, hypergeometric P values reported) for differentially expressed genes common between KTM-fed animals and animals depleted of DAF-2::AID in the indicated tissues using the auxin degron system [72]. Values >1 indicate over-enrichment, or that the same genes tend to be differently expressed in both animals consuming KTMs and animals depleted of DAF-2 compared to random chance. The overlap between differentially expressed genes that are either (H) up-regulated or (I) down-regulated in animals consuming KTMs and animals depleted DAF-2::AID in the intestine (hypergeometric P values are shown). (J) Representative fluorescent images (scale bar, 500 μm) and (K) quantification of the acyl-CoA dehydrogenase Pacdh-1::GFP reporter on the indicated microbial diets (n = 40, mean ± SD, ****, P<0.0001, ns, not significant, one-way ANOVA). Raw data underlying panels A-I and K can be found in S12 Data. (TIF) [file pgen.1011003.s008.tif]

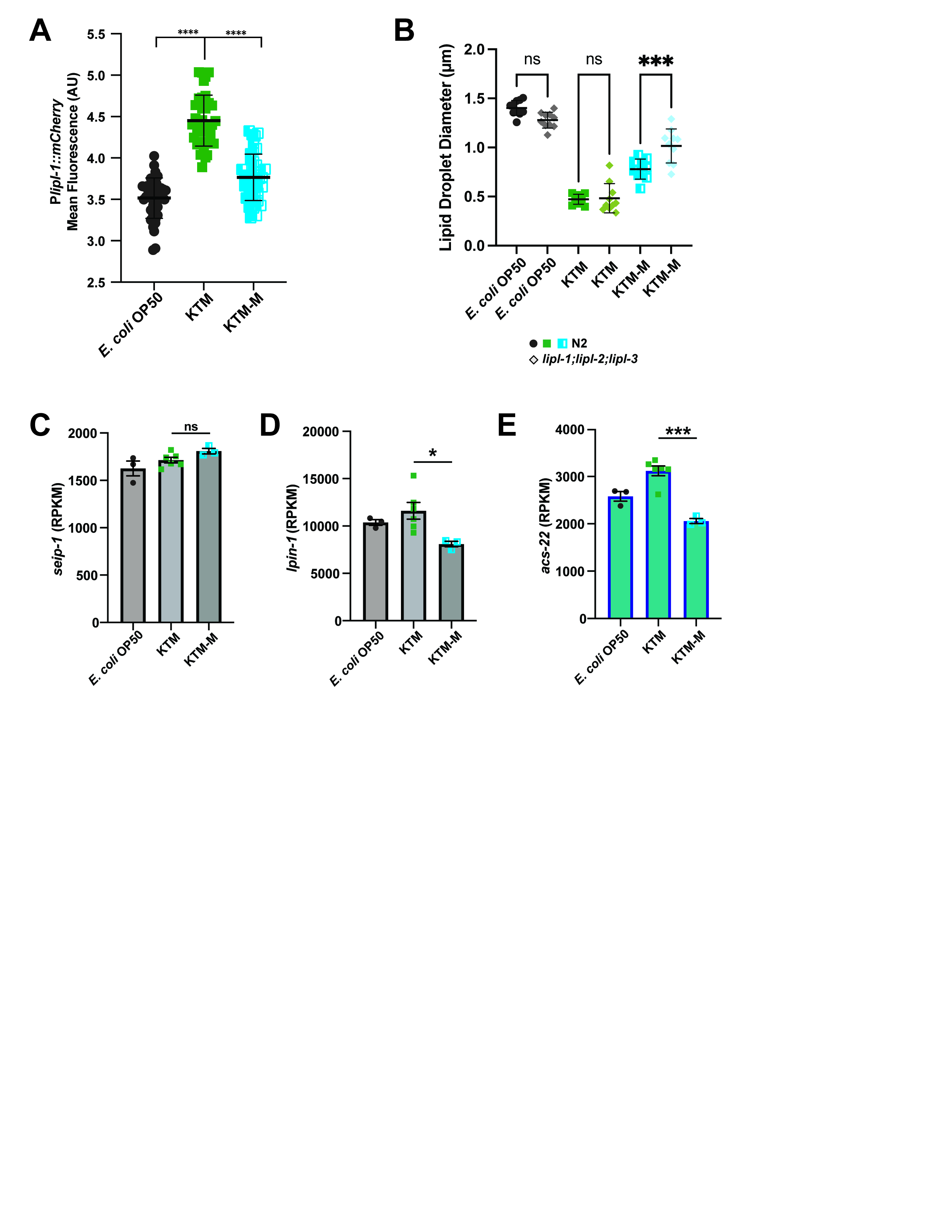

Supplement: S9 Fig — (A) Quantification of the expression levels of the lysosomal lipase Plipl-1::mCherry reporter in animals grown on E. coli OP50, KTM, and KTM-M (n>200, mean ± SD, ****, P<0.0001, one-way ANOVA). (B) Lipid droplet size measurements in wild-type N2 and lipl-1(tm1954) lipl-2(ttTi14801) lipl-3(tm4498) mutant animals with each datapoint representing the average intestinal lipid droplet diameter for a single animal (mean ± SD, ***, P<0.001, ns, not significant, one-way ANOVA). (C-E) Normalized gene expression values for the indicated TAG synthesis genes (mean ± SEM, ***, P<0.001, *, P<0.05, ns, not significant, one-way ANOVA). Raw data underlying panels A-E can be found in S13 Data. (TIF) [file pgen.1011003.s009.tif]
