# Supplementary material for: Kombucha Tea-associated microbes remodel host metabolic pathways to suppress lipid accumulation
Source: PLoS Genet. 2024 Mar 28;20(3):e1011003. doi: 10.1371/journal.pgen.1011003 (PMC10977768; doi:10.1371/journal.pgen.1011003)
Supplement: S2 Table — (PDF) [file pgen.1011003.s011.pdf]

Komagataeibacter:

Taxonomy	Strain	Genome Size	Covered Bases	Alignment Rate	% Alignment
Komagataeibacter eurupaeus	LMG18890	4227398	1696277	0.401257937	40.1257937
Komagataeibacter eurupaeus	SRCM101446	3797909	1572811	0.41412551	41.412551
Komagataeibacter intermedius	TF2	3883532	1607370	0.413893847	41.3893847
Komagataeibacter intermedius	AF2	4465062	1837864	0.411609962	41.1609962
Komagataeibacter intermedius	ENS15	3869279	1695425	0.43817595	43.817595
Komagataeibacter medellinensis	ID13488	3381856	2062677	0.60992455	60.992455
Komagataeibacter nataicola	RZS01	3760301	1408265	0.374508583	37.4508583
Komagataeibacter nataicola	LMG1536	3672972	1376887	0.374869996	37.4869996
Komagataeibacter oboediens	SI3053	3666777	1457638	0.397525674	39.7525674
Komagataeibacter oboediens	IMBG311	3732509	1482448	0.397171983	39.7171983
Komagataeibacter rhaeticus	**LMG_22126**	**3465200**	**3258204**	**0.940264343**	**94.0264343**
Komagataeibacter rhaeticus	**K15**	**4028747**	**3637660**	**0.902925897**	**90.2925897**
Komagataeibacter rhaeticus	**iGEM**	**3867346**	**3793802**	**0.980983341**	**98.0983341**
Komagataeibacter rhaeticus	**ENS9b**	**3693173**	**3647521**	**0.987638814**	**98.7638814**
Komagataeibacter rhaeticus	**9a1a**	**3644828**	**3595669**	**0.986512669**	**98.6512669**
Komagataeibacter rhaeticus	**AF1**	**3939137**	**3397907**	**0.862601885**	**86.2601885**
Komagataeibacter saccharivorans	**JH1**	3727857	1110676	0.29793954	29.793954
Komagataeibacter saccharivorans	**CV1**	3768311	1260929	0.334613836	33.4613836
Komagataeibacter xylinus	**E25**	3905082	1723402	0.441322871	44.1322871
Komagataeibacter xylinus	**DSM2325**	3727795	1600045	0.429220223	42.9220223
Komagataeibacter xylinus	CGMCC17276	3983026	1503780	0.377547121	37.7547121
Komagataeibacter xylinus	**CGMCC2955**	**3563314**	**3448064**	**0.967656513**	**96.7656513**

Acetobacter:

Taxonomy	Strain	Genome Size	Covered Bases	Alignment Rate	% Alignment
Acetobacter aceti	JCM20276	3967892	238692	0.060155871	6.01558712
Acetobacter aceti	NBRC14818	3693126	184304	0.049904607	4.99046066
Acetobacter aceti	TMW21153	3725037	108535	0.029136623	2.91366233
Acetobacter ascendens	LMG1590	2999217	356952	0.119015063	11.9015063
Acetobacter ascendens	LMG1591	3216032	421262	0.130988124	13.0988124
Acetobacter ascendens	SRCM101447	3285832	237506	0.072281845	7.22818452
Acetobacter ghanensis	LMG23848	2794032	135126	0.048362367	4.83623666
Acetobacter ghanensis	LMG23848T	2843936	213880	0.075205631	7.52056305
Acetobacter orientalis	FAN1	3214967	224644	0.069874434	6.98744342
Acetobacter orientalis	R82822	3291238	234104	0.071129466	7.11294656
Acetobacter orientalis	R83288	2969719	182429	0.061429718	6.14297178
Acetobacter oryzoeni	dm	3127455	261096	0.083485134	8.34851341
Acetobacter oryzoeni	SLV7	3106397	247707	0.079740935	7.97409346
Acetobacter oryzoeni	B6	3153180	319355	0.101280295	10.1280295
Acetobacter oryzoeni	R80287	3091914	285360	0.092292347	9.22923471
Acetobacter pasteurianus	386B	3078647	253486	0.082336819	8.23368187
Acetobacter pasteurianus	CICC22518	3056995	211889	0.069312838	6.93128383
Acetobacter pasteurianus	IFO_3283_ substrIFO3283_03	3339669	312717	0.093637124	9.36371239
Acetobacter persici	Ai	3361032	385182	0.114602301	11.4602301
Acetobacter persici	Dm46	3405333	485188	0.142478871	14.2478871
Acetobacter persici	Dm49	3378261	403718	0.11950468	11.950468
Acetobacter persici	TMW21084	3756676	445489	0.118585952	11.8585952
Acetobacter senegalensis	**108B**	**3969909**	**3335044**	**0.840080717**	**84.0080717**
Acetobacter senegalensis	**GYC27**	**3911037**	**3323362**	**0.84973934**	**84.973934**
Acetobacter tropicalis	**BDGP1**	**4139662**	**3282486**	**0.792935752**	**79.2935752**
Acetobacter tropicalis	**NBRC101654**	**3718538**	**3255668**	**0.87552366**	**87.552366**
Acetobacter tropicalis	CS_006_W139	3805032	2156700	0.566802066	56.6802066
Acetobacter tropicalis	DmPark25_167	3722678	2153925	0.57859557	57.859557
Acetobacter tropicalis	CS_006_W138	3801241	2156011	0.567186085	56.7186085
Acetobacter tropicalis	DmW_042	3838696	2197750	0.572525149	57.2525149
Acetobacter tropicalis	LMG1663	3769631	2291120	0.607783627	60.7783627
Acetobacter tropicalis	LMG19825	3557452	2183549	0.613795773	61.3795773
Acetobacter tropicalis	NBRC16470_v1	3532343	2175243	0.615807412	61.5807412
Acetobacter tropicalis	NBRC16470_v2	3675727	2208754	0.600902624	60.0902624

Zygosaccharomyces:

Taxonomy	Strain	Genome Size	Covered Bases	Alignment Rate	% Alignment
Candida boidinii	JCM9604	19377211	2387	0.00012319	0.01231859
Candida boidinii	M7017B	18755401	8398	0.00044776	0.04477644
Candida boidinii	NDK27A1	18791129	8305	0.00044196	0.04419639
Candida boidinii	NRRLY2332	19278657	8617	0.00044697	0.0446971
Candida boidinii	UNISSCb60	18794311	10275	0.00054671	0.0546708
Candida sake	CBA6005	14118184	71788	0.00508479	0.50847899
Candida sake	H14-1-4C	13310172	5533	0.0004157	0.04156971
Hanseniaspora valbyensis	NRRLY1626	11464036	6062	0.00052878	0.05287841
Lachancea fermentati	CBS6772	10264457	45394	0.00442245	0.44224453
Lachancea fermentati	M7023	10160833	46357	0.00456232	0.45623228
Pichia membranifaciens	KS471	11394881	12739	0.00111796	0.11179581
Pichia membranifaciens	NRRLY2026	11582150	9251	0.00079873	0.07987291
Saccharomyces cerevisiae	BY4742	12165468	138959	0.01142241	1.1422413
Saccharomyces cerevisiae	CICC1445	12069106	182503	0.0151215	1.51215011
Saccharomyces cerevisiae	S288C	12071326	117588	0.0097411	0.97411005
Schizosaccharomyces pombe	972h	12591251	15340	0.00121831	0.12183063
Schizosaccharomyces pombe	FLODUT	12813332	56762	0.00442992	0.44299172
Schizosaccharomyces pombe	JB4	12747577	31875	0.00250048	0.25004752
Saccharomyces uvarum	CBS7001	11854537	44432	0.0037481	0.37481008
Saccharomyces uvarum	CR10E1	11952332	39841	0.00333332	0.33333244
Saccharomyces uvarum	FM1277	11711626	29503	0.00251912	0.25191207
Saccharomyces uvarum	ZP964	11715280	42839	0.00365668	0.36566774
Zygosaccharomyces bailii	**CLIB213**	**10268813**	**8921769**	**0.86882184**	**86.8821839**
Zygosaccharomyces bailii	**ISA1307**	**21141148**	**16307734**	**0.7713741**	**77.1374099**
Zygosaccharomyces bailii	**IST302**	**10772966**	**9106309**	**0.84529265**	**84.5292652**
Zygosaccharomyces bisporus	NRRLY12626	10605989	509901	0.0480767	4.80767046
Zygotorulaspora florentina	NRRLY1560	11069688	44034	0.00397789	0.39778899
Zygosaccharomyces rouxii	ATCC42981	20910059	184392	0.00881834	0.88183395
Zygosaccharomyces rouxii	CBS732	9764635	89081	0.00912282	0.91228192
Zygosaccharomyces parabailii	**ATCC60483**	**20864403**	**16171272**	**0.77506517**	**77.5065167**
