# Supplementary material for: Kombucha Tea-associated microbes remodel host metabolic pathways to suppress lipid accumulation
Source: PLoS Genet. 2024 Mar 28;20(3):e1011003. doi: 10.1371/journal.pgen.1011003 (PMC10977768; doi:10.1371/journal.pgen.1011003)
Supplement: S3 Table — The strain names, genotypes, and associated references are shown. (PDF) [file pgen.1011003.s012.pdf]

**Strain**	**Genotype**	**Reference/Source**
N2	Wild-type *C. elegans*	[1], CGC
JU1212	Wild-type *C. elegans,* natural isolate	[2], CGC
MY10	Wild-type *C. elegans,* natural isolate	CGC
PB2801	Wild-type *C. brenneri*	CGC
PB4641	Wild-type *C. remanei*	CGC
AF16	Wild-type *C. briggsae*	CGC
DLS748	*rhdSi53[*P*lipl-1::mCherry::unc-54 3'UTR + cb-unc-119(+)] II; unc-119(ed3) III*	This study
DLS840	*lipl-2(rhd282[A423*]) lipl-1(rhd279[A391*]) V*	This study
DLS912	*lipl-3(tm4498) lipl-2(rhd282) lipl-1(rhd279) V*	This study
DLS913	*lipl-2(ttTi14801) lipl-1(tm1954) V*	This study
DLS952	*lipl-3(tm4498) lipl-2(ttTi14801) lipl-1(tm1954) V; ldrIs1[*P*dhs-3::dhs-3::GFP + unc-76(+)]*	This study
BOX213	*erm-1(mib15[erm-1::eGFP]) I*	[3], CGC
BCN9071	*vit-2(crg9070[vit-2::gfp]) X*	[4], CGC
DA465	*eat-2(ad465) II*	[5], CGC
GMW0020	*lipl-3(tm4498) lipl-2(ttTi14801) lipl-1(tm1954) V*	Dr. Eyleen O'Rourke Lab
GR1395	*mgIs49[*P*mlt-10::GFP::PEST + ttx-3::GFP] IV*	[6], CGC
LIU1	*ldrIs1[*P*dhs-3::dhs-3::GFP + unc-76(+)]*	[7], CGC
VL749	*wwIs24[*P*acdh-1::GFP + cb-unc-119(+)]*	[8], CGC
VS29	*hjSi56[*P*vha-6::3xFLAG::TEV::GFP::dgat-2::let-858 3'UTR] IV*	[9], CGC

References

1. Brenner S. The genetics of Caenorhabditis elegans. Genetics. 1974;77: 71–94. doi:10.1093/genetics/77.1.71

2. Andersen EC, Gerke JP, Shapiro JA, Crissman JR, Ghosh R, Bloom JS, et al. Chromosome-scale selective sweeps shape Caenorhabditis elegans genomic diversity. Nat Genet. 2012;44: 285–290. doi:10.1038/ng.1050

3. Sepers JJ, Ramalho JJ, Kroll JR, Schmidt R, Boxem M. ERM-1 Phosphorylation and NRFL-1 Redundantly Control Lumen Formation in the C. elegans Intestine. Front Cell Dev Biol. 2022;10: 769862. doi:10.3389/fcell.2022.769862

4. Perez MF, Francesconi M, Hidalgo-Carcedo C, Lehner B. Maternal age generates phenotypic variation in Caenorhabditis elegans. Nature. 2017;552: 106–109. doi:10.1038/nature25012

5. Raizen DM, Lee RY, Avery L. Interacting genes required for pharyngeal excitation by motor neuron MC in Caenorhabditis elegans. Genetics. 1995;141: 1365–1382. doi:10.1093/genetics/141.4.1365

6. Meli VS, Osuna B, Ruvkun G, Frand AR. MLT-10 defines a family of DUF644 and proline-rich repeat proteins involved in the molting cycle of Caenorhabditis elegans. Mol Biol Cell. 2010;21: 1648–1661. doi:10.1091/mbc.e08-07-0708

7. Zhang P, Na H, Liu Z, Zhang S, Xue P, Chen Y, et al. Proteomic study and marker protein identification of Caenorhabditis elegans lipid droplets. Mol Cell Proteomics. 2012;11: 317–328. doi:10.1074/mcp.M111.016345

8. MacNeil LT, Watson E, Arda HE, Zhu LJ, Walhout AJM. Diet-induced developmental acceleration independent of TOR and insulin in C. elegans. Cell. 2013;153: 240–252. doi:10.1016/j.cell.2013.02.049

9. Xu N, Zhang SO, Cole RA, McKinney SA, Guo F, Haas JT, et al. The FATP1–DGAT2 complex facilitates lipid droplet expansion at the ER–lipid droplet interface. Journal of Cell Biology. 2012;198: 895–911. doi:10.1083/jcb.201201139
