# Supplementary material for: Kombucha Tea-associated microbes remodel host metabolic pathways to suppress lipid accumulation
Source: PLoS Genet. 2024 Mar 28;20(3):e1011003. doi: 10.1371/journal.pgen.1011003 (PMC10977768; doi:10.1371/journal.pgen.1011003)
Supplement: Attachment — Submitted filename: Response_to_Reviews.docx [file pgen.1011003.s027.docx]

**Reviewer #1:**

*In this manuscript, DuMez-Kornegay et al. characterize the utility of a C. elegans model system in studying the impact of kombucha-tea associated microbes (KTMs) on organismal physiology. The manuscript is very well-written and extremely easy to follow, and the thoroughness of the experimental methodology and data analysis is commendable. The data is quite strong and well-controlled, and the model system is very interesting. I have very few comments about the study, all of which can be addressed textually. While I recommend a few experiments, these are all merely suggestions as the rigor of the data is sufficient that new experiments do not need to be performed, although in some cases (if the authors find it helpful) may benefit the strength of the manuscript.*

*1. My only main concern of the study is that the authors very elegantly state in their introduction and conclusion that the benefit of producing this model is to better understand the impact of KTMs on physiology – something that is hard to do in humans. However, other than making very strong claims on the fact that KTMs do not impact overall organismal health, there is very little done to study the benefits of KTMs. Based on what the authors argue is the strength of making this model, this is a glaring weakness. Perhaps the authors could benefit by performing a few physiological assays to determine whether a KTM diet is actually beneficial to some readout of health: healthspan (e.g., motility, gut barrier integrity, etc.), lifespan, stress resilience, etc. I don’t expect the authors to do a comprehensive battery of physiological assays, so this can be minimally addressed by using the RNA-seq analysis to determine whether transcriptional profiles generally associated with increased health/longevity are found to be differentially expressed in their animals.*

We thank the reviewer for their comments and agree that a physiological assessment of healthspan and lifespan in response to a KTM diet would be valuable; however, this would require several detailed phenotypical and mechanistic analyses, and thus, is outside of the scope of our current study. We did, however, address the reviewer’s curiosities by leveraging our RNA-seq data as suggested. We determined whether the genes that are differentially expressed in response to a KTM diet are also differentially expressed upon loss of *daf-2*, which results in increased stress resistance, improved healthspan, and extended lifespan. When DAF-2::AID is depleted in either germline, intestinal, muscle, or neuronal tissues, the genes that are misexpressed share significant overlap with the genes differentially expressed in response to a KTM diet. Thus, animals consuming the KTM diet, and to some extent the KTM-M diet, are likely to display increased health- and lifespan phenotypes since they have a similar transcriptional profile to animals lacking DAF-2; however, a rigorous mechanistic analysis will be necessary to determine the health benefits of KTM consuming in *C. elegans*.

The following was added to the text (lines 378-384): “Since misexpression of core metabolic genes alters longevity and stress resistance pathways [67,68], we queried whether these same genes were also misexpressed in animals with reduced levels of DAF-2 (i.e., the insulin receptor), which results in increased stress resistance, improved healthspan, and extended lifespan [69,70]. Indeed, depletion of DAF-2 in different tissues [70], including the intestine, results in transcriptional changes that are consistent with those seen in KTM-fed animals (Figure S6G). Together, these data suggest that consumption of fermenting KT microbes may remodel host lipid metabolism and stress resilience pathways to restrict fat accumulation and improve heathspan.”

*2. The reproduction phenotype is sort of glossed over as being only “minor” effect, but it is still a statistically significant decrease. Generally, a decrease in brood size is considered a negative effect, yet the idea of KTMs is that there is some “health benefit”. The authors really need to address this. Perhaps try to determine why is there a decrease in brood: are the health of the progeny compromised due to the decrease in somatic fat (this can decrease available nutrients given to progeny). Is there a decrease in yolk transmitted to progeny (VIT-2::GFP)? Is this associated with the decrease in germline genes they see?*

Since there is a modest reduction in brood size when animals consume KTMs, we agree that it is important to determine whether the decrease in somatic lipids restricts the mother’s ability to provision the next generation, thereby reducing the number of offspring. Thus, we measured the amount of yolk transmitted to embryos in animals expressing an endogenously tagged VIT-2::GFP protein. We found that yolk levels are slightly elevated in KTM-fed animals relative to *E. coli*-fed animals, which is consistent with elevated *vit-2* mRNA levels in KTM-fed animals (mRNA-Seq). These two figures have been added to Figure 3 (J and K) and Figure 3J in the previous version of the manuscript was moved to the supplement.

We have added a description of these results to the text (lines 243-250): “Thus, the ~20% reduction in fertility for KTM-fed animals is inconsistent with the more severe reduction in brood size of CR animals. It could, however, be consistent with impaired maternal provisioning of lipid-rich yolk to oocytes from intestinal fat stores, a process termed vitellogenesis. Thus, we next examined the mRNA levels of *vit-2*, which encodes a vitellogenin protein that mediates the intestine-to- oocyte transport of lipids, finding that *vit-2* levels are increased in animals fed a KTM diet compared to E. coli-fed animals (Figure 3J). Consistently, vitellogenin protein levels, which we measured in early embryos (prior to the 44-cell stage) using an endogenously tagged VIT-2::GFP protein, were also elevated in KTM-fed animals (Figure 3K), further substantiating that KTM consumption does not impair maternal lipid provisioning.”

Additionally, the reviewer asks if there is a decrease in germline gene expression; however, we observed little difference in the expression of reproduction-related genes when we compared KTM and *E. coli* OP50-fed animals (Figure 3L) or any other diet (Figure 3M-N). If the reviewer is referring to data from our mRNA-Seq enrichment analysis displayed in Figure 5D, these results do not suggest a decrease in germline genes, but rather that there are less germline genes differentially expressed than expected by random chance (fold enrichment = observed/expected). We have described this method in detail in the methods section. Thus, germline differential gene expression in KTM-fed animals is under-enriched, further indicating that reproduction is not negatively impacted by KTM consumption.

Nonetheless, we have clarified this statement in the main text (lines 391-393):

“These data indicate that while genes expressed in the intestine are commonly differentially expressed in animals consuming KTMs, genes expressed in other tissue types tend not to be differentially expressed in KTM-fed animals.”

and the legend for Figure 5:

“Values <1 indicate that genes expressed in the indicated tissue type tend not to be differentially expressed (under-enriched), while values >1 indicate tissues where differential expression is more common than expected by random chance (over-enriched).”

*3. The authors should at least comment in text about the decrease in desaturate expression, which is normally correlated with increase in specific fats, yet they see an overall decrease in fats (likely due to the increased expression of other lipases, etc.).*

The reviewer makes an excellent point regarding the differential expression of several desaturase genes in response to KTM consumption. We agree that changes in the lipid profiles when animals consume KTMs is likely important to their physiology, however, measurements of specific lipid species in KTM-fed animals (by GC-MS), as well as the follow up mechanistic studies to provide context of how these specific lipids regulate organismal physiology, represents an dramatic extension of our current study, and thus, we believe are outside of the scope of the work presented here.

We have addressed this point in the Discussion (lines 486-501):

“To gain a comprehensive view of the host metabolic response to Kombucha, we performed mRNA sequencing of animals consuming KTMs. While expression of developmental or reproduction genes were globally unchanged, expression of numerous lipid metabolism genes were specifically altered in response to KTMs, with a strong enrichment for genes known to function in the intestine. These include gene products that function in various aspects of lipid biology, including β-oxidation of lipids (*acdh-1* and *acdh-2*), fatty acid desaturation (*fat-5* and *fat-7*), triglyceride synthesis (*dgat-2*), and lipolysis (*lipl-1*, *lipl-2*, and *lipl-3*). The stearoyl-CoA desaturase genes, in particular *fat-5* and *fat-7*, were down-regulated in KTM-fed animals. This finding is notable since the *C. elegans* desaturases have lipid substate preferences, and thus, differential expression of individual *fat* genes can result in alterations in the abundance of specific monounsaturated or polyunsaturated fatty acids [85]. FAT-5, which desaturates palmitic acid (16:0) to generate palmitoleic acid (16:1n-7), is transcriptionally down-regulated in KTM-fed animals, possibly resulting in a decrease in palmitoleic acid and increase in palmitic acid or other unsaturated fatty acids that are derived from palmitic acid. Specific changes in the abundance of monounsaturated or polyunsaturated fatty acids may contribute to the fasting-like state displayed by KTM-fed animals; however, lipidomic studies, paired with fatty acid supplementation experiments and genetic analyses, will be needed to resolve the role of the *C. elegans* desaturases in mediating the host response to KTM consumption.”

*4. Is it concerning that the authors see a decrease in neuron-related genes? Is neuronal function affected in these animals (e.g., behavior assays, chemotaxis, etc.)? Again, this can be addressed textually, and I don’t think actual experiments are necessary.*

As described above (point #2), values >1 in our enrichment analysis (Figure 5D) indicates that differential expression in that tissue is greater than expected by random chance, while values <1 indicate less differential expression than expected by random chance. We have clarified this in the main text and in the figure legend (see the changes described in point #2). Moreover, based on our behavioral assays (*i.e.*, lawn avoidance and food choice assays), animals consuming KTMs do not show neuronal impairment. Thus, we do not expect that neuronal function is affected in these animals.

**Reviewer #2:**

*Dowen and colleagues present a research study that examines how microbes that are found in kombucha influence C. elegans. I commend the authors on an outstanding study that is well designed and with appropriate experimental controls. I found the manuscript clear to read and appreciate that the data was presented matter-of-factly and not over sold. In light of the popularity of kombucha to the general public, I believe this study will be of general interest beyond the scientific community. Moreover, this work expands upon the list of beneficial microbes that can influence organismal physiology. In my opinion, this work is a perfect fit for PLOS Genetics and can be accepted in its current form. I do note some VERY minor concerns that could help with readability, but these are not required and I leave it to the editor and author if they are helpful.

1. I would encourage the authors to discuss all data points that reach statistical significance in their work as the collectively might contribute to the overall outcomes. This does not require a significant addition to the discussion, but there are several weak phenotypes (but significant) that are not thoroughly accounted for and might be interesting.*

We thank the reviewer for their insightful comments and we acknowledge that there are several comparisons that reach statistical significance presented in the figures that are not discussed in depth in the manuscript. For example, we regularly performed statistical comparisons between *E. coli* OP50, *E. coli* HT115, and *A. tropicalis*-fed animals; however, we have chosen to prioritize discussing only those conditions relative to KTM-fed animals, as this is the focus of the manuscript. We believe that a detailed discussion of these comparisons might detract from our primary points regarding Kombucha-associated microbes, and thus, we would prefer to allow the reader to draw their own conclusions for these minor points. Lastly, the phenotypical differences between *E. coli* OP50 and HT115 (or *A. tropicalis*) have either been described previously or, we believe, are outside of the scope of this study and will be suitable for further characterization (and discussion) in a follow-up study.

*2. It would be useful to better exploit the RNAseq data to identify classes of genes that have been found to impact organismal healthspan. These can be done with publicly available tools in WormBase.*

We agree with the reviewer and have leveraged our mRNA-Seq data to compare the genes altered in KTM-fed animals to those genes altered by DAF-2 depletion. The details of these analyses are described above (Review 1, point 1).

**Reviewer #3:**

*In this manuscript by DuMez-Kornegay et al., the authors present the findings that consumption of the microbes found in Kombucha Tea, a popular fermented beverage with purported health benefits, by the nematode C. elegans leads to reduction in fat mass. The authors report that reductions in fat mass are accompanied by increases in lysosomal lipase expression and reduced expression of enzymes of fatty acid biosynthesis, leading them to speculate that lipid droplet catabolism by lipophagy is important for the observed changes in overall lipid stores, in parallel with reductions in lipid synthesis.

Major:
1. Pumping has been shown to be a poor corollary of food intake. At least the authors should acknowledge this; at best they could assess uptake of a vital dye such as C1-BODIPY-C12.*

We appreciate the reviewer’s comments and agree that pumping rate do not always correlate with food intake or nutrient absorption. Therefore, we assessed food uptake and nutrient absorption using the vital dye C1-BODIPY-C12 and found that KTM-fed animals consume the dye and absorb it from the intestinal lumen. We have included these data in Figure 4 and Supplemental Figure 6.

We have added a discussion of these results to the main text (lines 346-352):

“Next, we assessed nutrient absorption in KTM-fed animals by supplementing the KTM lawn with the vital dye C1-BODIPY-C12, which is consumed with the food and can readily cross the intestinal apical membrane. Following a three-hour pulse of BODIPY, animals consuming *E. coli* OP50, KTM, and KTM-M all have detectable levels of BODIPY in their intestinal epithelial cells (Figure 4K and S6). Since animals consuming KTMs have very few, small lipid droplets the BODIPY staining was diffusely distributed throughout the intestinal cells; however, in *E. coli* OP50 and KTM-M-fed animals the dye localized to intestinal lipid droplets.”

*2. Because of the major intestinal colonization observed with KTM, the authors should test whether the intestine is involuted and/or the luminal volume is expanded. This could be consistent with an increase in autophagic activity and loss of lipid droplets. Also, is the intestine colonized by bacteria or yeast or both?*

We agree with the reviewer that this is an important point to consider. To assess whether the intestinal lumen volume is altered in KTM-fed animals, we used a strain expressing ERM-1::GFP, which localizes to the apical membrane of intestinal cells and facilitates luminal diameter measurements. These experiments revealed that animals consuming KTMs do have an extended lumen, as expected based on the robust microbial colonization of the lumen by both bacteria and yeast KTMs (Figure 1F-G and S5I-K), but we could not detect any involution of the intestine.

We have added a discussion of these results to the main text (lines 335-342):

*“*To further assess the ability of the KT microbes to colonize the gut, we quantified the intestinal lumen size of animals reared on the *E. coli* OP50, KTM, and KTM-M diets. Using animals expressing ERM-1::GFP, which localizes to the apical surface of intestinal cells and facilitates luminal measurements, we found that individuals consuming a KTM diet had an increased intestinal lumen diameter compared to animals consuming *E. coli* OP50 but not the KTM-M diet, suggesting that any diet containing KT microbes stimulates intestinal bloating (Figure S5K).

The presence of *Z. bailii* in the gut, which may contribute to intestinal bloating, raised the possibly that the yeast (or the other KT microbes) may restrict nutrient absorption, resulting in caloric restriction…*”*

*3. Pathogen exposure and genetic activation of the stress transcription factor skn-1 have been shown to dramatically reduce somatic fat, much like the effects observed here. The authors should test whether this is a pathogenic response as well as the dependency upon skn-1. The RNAseq data in figure 5 also indicate enrichment of a lipid and pathogen gene expression signature, making this possibility even more likely.*

As pointed out by the reviewer, SKN-1 is known to regulate the expression of lipid metabolism genes and coordinate metabolic states under different physiological contexts. Defining the role of SKN-1 in mediating the host metabolic response to KTM consumption is prudent; however, this would require an in-depth mechanistic study to establish the tissue of action and the mode of regulation. Furthermore, these studies would not alter any of the conclusions described in our current manuscript. Thus, we believe that these experiments are better suited for a follow-up investigation.

To investigate the pathogen response in KTM-fed animals, we looked more closely at our mRNA-Seq data. The GO ontology enrichment analysis that appears in Figure 5C was conducted with a small set of genes – the 295 KTM-specific genes – and only 10 of these genes were characterized as “Stress Response: Pathogen” (WormCat, see figure below). While this represents a statistically significant enrichment, it is a small set of genes relative to the other categories. Of those 10 innate immunity genes, six are down-regulated (see below). Additionally, KTM-fed animals do not display a lawn-leaving behavior that suggestive of a pathogen response (Figure 1D). Thus, it is unlikely that there is a KTM-specific pathogen response that is responsible for the metabolic phenotypes seen in KTM-fed animals. It is likely, however, that changes in specific metabolites may influence the expression of innate immunity genes, as has been recently demonstrated for phosphatidylcholine (Fanelli, Sci Adv 2023).

*4. The authors should be sure statistical tests used are indicated in each legend, as well as statistical thresholds (e.g. missing in Fig. 1D).*

The statistical test and thresholds have now been reported for Figure 1D.

*5. The lipid quantification by oil red O indicates an extremely small difference in intensity (Y axis scaling should be from 0 not 6.20 to 6.40), whereas the differences seen by Nile red, biochemical quantification, or lipid droplet analysis indicate a much larger effect. The authors should explain the reason for the differences and perhaps consider or explain why the results are so quantitatively discordant.*

Our initial lipid abundance studies of KTM-fed animals employed four different approaches – Oil Red O staining, Nile Red staining, biochemical triglyceride measurements, and lipid droplet measurements – that all yielded identical results, demonstrating that neutral lipids are reduced upon KTM consumption. We found Oil Red O staining to be highly reproducible when analyzing our KTM-fed animals and we have leveraged that approach to tease out the host response to the individual components of the KT culture and for comparisons between fermenting and non-fermenting mixes (Figure 4). Again, these results were highly reproducible. Our additional Oil Red O studies were generally performed in parallel to other lipid assays (Figure 2, 6). Thus, we are confident that our Oil Red O measurements are a true reflection of host lipid levels.

The values shown on the Y axis of our Oil Red O quantification plots are arbitrary units. An individual data point in these figures represents the average gray values for a single animal, which includes regions that are stained with Oil Red O and those that are not. Unstained regions are not as dark as stained regions but are significantly darker than background (areas surrounding the animal). Therefore, the unstained regions within animals inflate the average and compress the data. Even if subtraction of this background was feasible (by selecting a subjectively defined “unstained” region), it would just change the values on the Y axis in a linear fashion. Therefore, we have chosen to perform minimal processing of the data and have simply reported the gray values.

We have now clarified this approach in the methods section (lines 697-704):

“For quantification of Oil Red O staining, whole animals were outlined using Fiji and the average gray value for each individual (0 to 65,536) was measured. The resulting values were subtracted from 65,536, which inverts the scale so that strongly stained animals now have higher values. True background values are the unstained regions within each animal; however, these regions are impossible to identify objectively. Thus, no background subtraction was performed, which compresses the data to a small range of values (55,000 to 65,000, which we report 5.5 to 6.5). We found this approach to be highly reproducible. For the intestinal Oil Red O staining analyses (Figures 6G and 6L), the mean gray values were calculated in a box (25x25 pixels) drawn within the first two intestinal cells and the analysis was performed as described above.”

*6. C. elegans accumulates much more lipid as they transit reproductive life; given that the KTM animals appear smaller, is body size/growth an explanation for the lower fat mass observed? It does not appear to be alterations in time to adulthood based upon data in Fig. 3. The authors should look at later days of adulthood.*

Looking at later days of adulthood, KTM-fed animals accumulated lipids over time in a manner similar to animals consuming the traditional laboratory food sources (*i.e.*, *E. coli* OP50 and *E. coli* HT115); however, lipid levels remained lower in KTM-fed animals at these additional timepoints. We agree with the reviewer that this observation adds additional context to our results, and thus, we have added these data to the manuscript.

We have also added a discussion of these results to the main text (lines 182-184):

“These trends continued during and after the reproductive period (Figure S3A), suggesting that KTMs restrict host lipid accumulation throughout reproduction and during the aging process.”

*7. Lipid droplet number in addition to size should be included in the analysis in Fig 2/S3, if the analysis was completed properly to objectively quantify lipid droplet number per HPF, for example.*

We agree with the reviewer that assessing LD abundance is important and should be included. Thus, we have performed this analysis and have found that LD number is decreased in KTM-fed animals. These results have been added to Figures 2/S6 and described in the results section (lines 196-202).

Also, see point #10 below.

*8. Filter sterilization may not be sufficient to remove all microbes/spores/bacterial products from the KTM-FM mix prior to introduction of the cultured bacteria. This should be addressed, experimentally if possible.*

To address the concern that filter sterilization may not be sufficient to remove environmental microbes/spores from the Kombucha Tea media (containing ~5% sucrose), we plated filtered KT media with and without KTMs on NGM plates and monitored growth for up to 14 days. No microbial growth was seen on the NGM plates seeded only with the filtered KT media after 14 days (see below). Thus, we are confident that the KT media used for the KTM-FM experiments has been properly filter sterilized. We acknowledge that this does not eliminate the possibility that bacterial products from the steeped tea are present within the plate media (in a diluted form); however, these metabolites are not sufficient to confer metabolic phenotypes in worms consuming the KTM-M diet.

The following text was added to the “NGM Kombucha Plates” method section (lines 605-607):

“To confirm that the filter sterilized tea media was free from microbes and/or spores, the sterilized tea media was plated on NGM plates and monitored for growth over 14 days, which resulted in plates free of microbial growth.”

*9. The “wash” experiments in fig 4H-I are informative but don’t entirely say that the small molecules are dispensable; rather they might be important for getting the bacteria to the state where they induce lipid depletion. This possibility should be indicated rather than dismissing the importance of these small molecules. Finally, are the authors certain that the post-fermented mix does not harbor other microbes that proliferated during the fermentation process? If filtration doesn’t completely sterilize the KTM-FM pre-fermentation mix, this could be a possibility. Sequencing could be used to rule this out.*

We agree with the reviewer that our results do not eliminate the possibility that tea-derived metabolites play a role in establishing the symbiotic culture. Therefore, we have modified the text on lines 319-324:

“Additionally, removal of the fully fermented KTM supernatant followed by repeated washes of the KTMs with a 5% sucrose solution prior to seeding the NGM plates did not alter host lipid accumulation in response to KTMs (Figures 4H and 4I), suggesting that the small molecules in the green and black tea may be dispensable for conferring host lipid phenotypes. This result, however, does not rule out the possibility that the tea-derived metabolites are crucial for establishing the symbiotic Kombucha culture.”

As described above in point #8, plating the filtered sterilized tea media on NGM plates failed to generate microbial colonies, suggesting that additional microbes are unlikely to be found in the KTM-FM samples.

*10. The oil red o data in figure 6G show extremely blunted effect sizes, as above. A more sensitive method should be considered for assessing changes in fat, even though the effects are significant; a 6% difference is of dubious significance (and this appears to be the magnitude based upon the scaling of the axis. Why are the oil-red-o data showing compression of effect size if the effects are really as dramatic as indicated by the lipid droplet reporter and nile red?*

To expand on our Oil Red O data shown in Figure 6G, we have examined lipid droplet size and lipid droplet number in the *lipl-1; lipl-2; lipl-3* triple mutant following KTM consumption. Loss of *lipl-1,2,3* increased lipid droplet number but not size, suggesting that the LIPL-1,2,3 proteins promote lipid droplet degradation in KTM-fed animals.

We have added these results to the main text (lines 433-439):

“Since the LIPL-1,2,3 proteins localize to lysosomes and catabolize LD-associated TAGs, we reasoned that LD size or quantity may be altered in *lipl-1,2,3* mutants consuming KTMs. Therefore, we crossed the DHS-3::GFP reporter into the *lipl-1(tm1954); lipl-2(ttTi14801); lipl-3(tm4498)* triple mutant and measured intestinal LDs. Triple mutant animals fed the KTM diet, but not the KTM-M diet, had more LDs compared to wild-type animals; however, the LD size was similar between wild-type and mutant animals (Figures 6H-J and S8B), suggesting that the LIPL-1,2,3 proteins promote LD degradation, but not LD shrinking, in KTM-fed animals*.”*

In the discussion we added the following text, which is underlined here (lines 532-536):

“It is possible that *dgat-2* expression is controlled by the same signaling networks that control the expression of the LIPL-1,2,3 lysosomal lipases, which together restrict the accumulation of lipids during KTM feeding. This could also explain why loss of *lipl-1,2,3* increases LD abundance but not size, as *dgat-2* likely remains down-regulated in these animals following KTM consumption.”

See point #5 above for a discussion on the Oil Red O staining.

*11. The leap from lysosomal lipases to lipophagy is not substantiated and could be speculated in the discussion but should not be indicated in the results. If the authors want to substantiate this claim they should test the requirement of classic autophagy genes, as the effects on lipid levels could be indirect through increased classic oxidation or reduced synthesis. Experiments could be done to indicate increased oxidation (oxygen consumption) or decreased synthesis (e.g. 13C labeling as in Perez and Van Gilst PMID 18762027) but those may be beyond the scope of this investigation. Instead the authors could present the data on expression and functional significance and speculate in the discussion rather than declaratively concluding “lipophagy” as the abstract and results suggest. In contrast the DGAT OE experiment is more convincing, but still the effect size is very small again because of the use of the oil red o assay.*

We appreciate the reviewer’s concerns and agree that our data is only suggestive that KTM consumption is triggering lipophagy. Therefore, we modified our language throughout the manuscript, particularly in the results and discussion sections. The relevant text is:

1. In the abstract, we say “…upregulation of a suite of lysosomal lipase genes that are induced during lipophagy”, which refers to previous studies and not our own. Thus, we think that the use of lipophagy is appropriate in this context in the Abstract.

2. In the introduction, we use “lysosomal lipases that function in lipophagy” again and speculate that “Our results suggest that Kombucha Tea consumption may alter lipid droplet dynamics by promoting their degradation via lipophagy”. We believe that use of “lipophagy” is appropriate in both cases.

3. In the results section, we have removed “likely through induction of lipophagy” (line 424) from the description of our results in Figure 6. Additionally, we have removed “via lipophagy” when describing Figure 6N (line 439). The other instances of “lipophagy” in the results section describe previous work on the *lipl* genes and are therefore appropriate in our view.

4. In the discussion section, our language is speculative, and we feel the use of “lipophagy” is warranted. We did add “may” to the following sentence (line 504): “Our discovery that *C. elegans* animals consuming a KTM diet may have elevated levels of lipophagy, and potentially a broader autophagy-driven metabolic reprograming, is consistent with these claims and suggests that future studies deconvoluting the host response to Kombucha consumption at the molecular level will provide insight into how Kombucha Tea may alter human metabolism.”

*Minor:
12. Introduction--the authors may want to cite direct consequences of reducing microbial diversity, e.g. C. difficile colitis in response to antibiotics.*

We appreciate this suggestion from the reviewer and agree that including an example that highlights the consequences of reducing microbial diversity is important. Thus, we have added the following text to the manuscript (lines 62-63):

“For example, *C. difficile* colitis can arise from antibiotic use and a subsequent loss of microbial diversity in the gut, resulting in severe gastrointestinal symptoms and potentially death [4]”

*13. Line 61 “hepatoprotective” is not a widely used term, the authors may want to expound on the meaning they intend.*

To clarify our use of the term “hepatoprotective” we have added the following text to line 56: “…improved hepatoprotective activity (*i.e.*, protection against liver toxins)…”.

*14. Rather than “standard C. elegans husbandry” the authors may wish to use “Axenic preparation of C. elegans cultures renders…”*

We thank the reviewer for this suggestion and have made the following edit to line 72: “Axenic preparation of *C. elegans* cultures renders these bacterivore animals microbe-free …”
 *15. The authors may want to specify that NGM-KTM plates contained no antibiotics or antifungals.*

This is a great suggestion as it reiterates the fact that we allow the KTMs to establish a community on the NGM plates. Therefore, we have added the following text on line 107: “…we first sought to establish a reproducible method to deliver KTMs to *C. elegans* animals via feeding on our standard agar-based nematode growth media (NGM), which do not contain any antibiotics or antifungals.”

*16. Line 224, 225, 228 ab libitum should be ad libitum.*

These typos have been fixed.

*17. It looks like KTM could disrupt the pulsatility of mlt-10 expression/turnover. Also the KTM data for 3A/B/C appear to be the same without replication and without statistics. How many times was this experiment done and were the results representative? How many animals were counted at each timepoint?*

We followed at least 24 animals per condition over the course of their development from L1s to Young Adults scoring the plates every hour for 70 hours. This was done at least twice for each food source except for KTM-M and KTM-FM as reflected in our methods (see below). The panels (3A/B/C) reflect 3 different comparisons of the KTM data (relative to each other food source) from one representative experiment. Placing all 4 curves in one plot would make the figure difficult to interpret. We have added this text to the legend for clarification: “A single representative experiment is displayed in panels A-C.”

This text is from our Methods section, which we have left unchanged:

“Between 1-8 synchronized *mgIs49[*P*mlt-10::GFP::PEST]* L1s were dropped into each well of a 24-well plate containing NGM media and seeded with 20 uL of each food source (one plate per food source). Animals were reared at 20°C and visualized by fluorescence microscopy every hour for 70 hours on a Nikon SMZ-18 Stereo microscope. At each time point, animals were scored as green (molting) or nongreen (not molting). Wells without animals were censored. The fraction of animals molting for each timepoint was calculated and plotted with Prism 9. The experiments were performed at least twice (except for KTM-M and KTM-FM) with similar results.”

We do observe peaks of MLT-10 expression in KTM-fed animals (Figure 3A) but agree that the MLT-10 expression valleys are not as extreme as the other food sources. It’s likely that there is some asynchrony in developmental rates, particularly during the L1/L2 stages, that can be resolved using this highly sensitive reporter; however, these data also show KTM-fed animals reach adulthood synchronously.

*18. The authors could consider a chi-squared statistic on Figure 3G to show significance.*

We appreciate the reviewer’s suggestion and have conducted a chi-squared analysis on the data presented in Figure 3G to determine whether the distribution of developmental ages are statistically different between *E. coli* OP50 vs KTM-fed animals. An analysis was individually performed for wild-type and *eat-2* mutant animals. The results from the chi-squared statistical analysis have added to Figure 3G and P values are reported in the legend.

*19. Did the authors expect to see changes in developmental genes at adulthood (Fig. 3D-F)? Why was an earlier timepoint not queried if differences in development were to be uncovered?*

We agree with the reviewer that our argument is flawed due to the age of animals that was analyzed; however, we believe that this analysis still adds value to the manuscript, as it demonstrates that there is not widespread developmental asynchrony (or heterochrony) within the population.

We have modified the text accordingly (lines 219-222):

“Upon inspection of 2,229 genes previously associated with *C. elegans* development [28], we observed very few gene expression differences between KTM-fed animals and those fed control diets (Figures 3D-F), suggesting that the KTM-fed population reaches adulthood synchronously.”

*20. The statement on lines 291-294 is not well justified by the 5xKTM experiment and should be altered or removed.*

We agree and have modified our language accordingly (see underlined) in the second half of the sentence (lines 297-300):

“Surprisingly, increasing the concentration of KTMs present in the lawn fivefold (5x KTM) further reduced lipid levels compared to our standard KTM lawn (Figures S5A-C), indicating that an increase in the microbial concentration, which likely results in additional available nutrients, does not increase host lipid accumulation.”
