# Supplementary material for: Kombucha Tea-associated microbes remodel host metabolic pathways to suppress lipid accumulation
Source: PLoS Genet. 2024 Mar 28;20(3):e1011003. doi: 10.1371/journal.pgen.1011003 (PMC10977768; doi:10.1371/journal.pgen.1011003)
Supplement: Attachment — Submitted filename: Response_to_Reviews.docx [file pgen.1011003.s028.docx]

**Reviewer #3:**

*The authors for the most part have satisfactorily addressed my concerns. Only a few very minor points and/or errors:

BODIPY staining except at high doses mostly stains gut granules. Many references show this e.g. Yen et al., PlosOne 2020, so the authors may want to adjust the statement 346-352 to indicate that acidified organelles and lipid droplets are illuminated by the BODIPY feeding.*

We absolutely agree with the reviewer’s comments and have made the following text edits on lines 350-352 (the underlined text below has been added):

“Since animals consuming KTMs have very few, small lipid droplets the BODIPY staining was diffusely distributed throughout the intestinal cells; however, in the *E. coli* OP50 and KTM-M-fed animals the dye localized to intestinal lipid droplets and lysosome-related organelles [53,67]”

The following reference has been added [67]:

Yen K, Le TT, Bansal A, Narasimhan SD, Cheng JX, et al. (2010) A Comparative Study of Fat Storage Quantitation in Nematode *Caenorhabditis elegans* Using Label and Label-Free Methods. PLOS ONE 5(9): e12810. <https://doi.org/10.1371/journal.pone.0012810>

*The legend for figure 4J has an error, it does not show images of Calcofluor, and there are 2 “K” references.*

The legend for Figure 4 has been corrected as follows:

“…(J) Quantification of Oil Red O staining of day 1 adult animals consuming *E. coli*, KTMs, and *Z. bailii* with or without dead *E. coli* supplementation as an inert nutrient source (mean ± SD, ****, *P*<0.0001, n.s., not significant, one-way ANOVA). (K) Representative DIC and fluorescence images of the intestine after feeding the indicated diets supplemented with C1-BODIPY-C12 (stars indicate the intestinal lumen and arrowheads indicate the intestinal epithelial cells; scale bars, 10 mm). Expanded data for panel A can be found in S2 Table and the raw data underlying panels C, E, F, G, I, and J can be found in S4 Data.” [lines 1154-1160]

*Strictly speaking the t-test is not an appropriate statistical test for figure 6A-E and 6J unless it is multiple hypothesis testing corrected. Meanwhile 6K cannot be one way anova because it has only 2 datasets.*

As the plots display RPKM (reads per kilobase of transcript per million mapped reads) values for *E. coli* OP50, KTM, and KTM-M fed animals, a one-way ANOVA is indeed more appropriate. Therefore, we have made the corresponding corrections to the figures, legends, and supplementary data files associated with Figures 6A-E, 6J, and S9C-E. Additionally, we have corrected the typographical error in the legend for Figure 6K as a T-test was indeed the statistical test applied to the two data sets in this experiment. [line 1190-1191]

*I suggest the authors look very carefully through their figure assignments and legends one last time.*

While making the above corrections we noticed additional typographical errors, which have now been corrected:

(1) For consistency, we removed the “.” from non-significance demarcations in Fig 4, Fig 6, Fig S4, and Fig S8, as well as in the corresponding legends, which now read as “ns” rather than “n.s.” in cases of no statistical significance.
(2) Corrected typographical error in legend for Figure S6A to reflect the statistical test performed, one-way ANOVA.
(3) Corrected typographical error in legend for Figure 2 E and H from “**, P<0.01” to “*, P<0.05”.
(4) Corrected the tab labels in supplementary data files to match the correct figure number.
(5) “*ab libitum”* corrected to “*ad libitum*” [line 232-233].
(6) Deleted a redundant “yeast” from line [262].
(7) Corrected “heathspan” to “healthspan”. [line 384].
